# Mifepristone Improves Octreotide Efficacy in Resistant Ectopic Cushing's Syndrome

**DOI:** 10.1155/2016/8453801

**Published:** 2016-02-17

**Authors:** Andreas G. Moraitis, Richard J. Auchus

**Affiliations:** ^1^Corcept Therapeutics, 149 Commonwealth Drive, Menlo Park, CA 94025, USA; ^2^Division of Metabolism, Diabetes, and Endocrinology, Department of Internal Medicine, University of Michigan, 1150 West Medical Center Drive, Ann Arbor, MI 48109, USA

## Abstract

A 30-year-old Caucasian man presented with severe Cushing's syndrome (CS) resulting from ectopic adrenocorticotropin syndrome (EAS) from a metastatic pancreatic neuroendocrine tumor. The patient remained hypercortisolemic despite treatment with steroidogenesis inhibitors, chemotherapy, and octreotide long-acting release (LAR) and was enrolled in a 24-week, phase 3 clinical trial of mifepristone for inoperable hypercortisolemia. After mifepristone was added to ongoing octreotide LAR treatment, EAS symptoms essentially resolved. Cortisol decreased dramatically, despite mifepristone's competitive glucocorticoid receptor antagonist effects. The clinical and biochemical effects reversed upon mifepristone discontinuation despite the continued use of octreotide LAR therapy. Substantial improvement in octreotide LAR efficacy with mifepristone use was noted in this patient with ectopic CS, consistent with upregulation of somatostatin receptors previously downregulated by hypercortisolemia.

## 1. Introduction

Chronic hypercortisolemia resulting from ectopic adrenocorticotropin hormone secretion (EAS) (nonpituitary) accounts for approximately 10% of all adrenocorticotropin- (ACTH-) dependent Cushing's syndrome (CS) [1, 2]. Although localized primarily in the chest, consisting of mainly bronchial (foregut) neuroendocrine tumors (NETs) and small-cell lung carcinoma, EAS is also associated with medullary thyroid carcinomas, gastrointestinal NETs, and thymic NETs, and, less frequently, other tumor types [2–4]. The primary treatment of EAS is surgical removal of the tumor when possible. If surgical resection is not possible or successful, medical therapy is necessary.

Depending on the etiology, EAS-associated tumors can express multiple somatostatin receptor subtypes (e.g., SST2, SST1, and SST5) [5]. This finding has enabled the use of somatostatin receptor scintigraphy (e.g., [^111^In]-pentetreotide or octreoscan and [^68^Ga]-octreotide-derivative positron emission tomography) for tumor localization and, in some cases, targeted treatment with somatostatin analog, octreotide [2, 4, 6–9], which has high affinity for SST2 [10]. However, octreotide therapy is frequently ineffective, limiting its utility as a therapeutic and diagnostic agent [11, 12].

Glucocorticoids have been shown to directly downregulate SST2 expression in human NET cells [13], as well as in human and murine corticotrope adenoma cells [14, 15]. The downregulation of SST2 in human neuroendocrine cell lines was found to be reversed with the addition of mifepristone, a glucocorticoid receptor (GR) antagonist [13]. Mifepristone may also directly influence tumoral SST2 expression levels in human NETs [16]. Medical therapy with mifepristone in 2 patients with EAS resulted in increased posttreatment uptake, positive octreoscan, and subsequent tumor localization [16]. In contrast, the capacity of mifepristone to enhance the clinical therapeutic efficacy of somatostatin analogs in reducing ACTH and cortisol production has not yet been demonstrated. We report a case of EAS demonstrating a synergistic effect of mifepristone in combination with octreotide long-acting release (LAR).

## 2. Case

**Figure 1:**
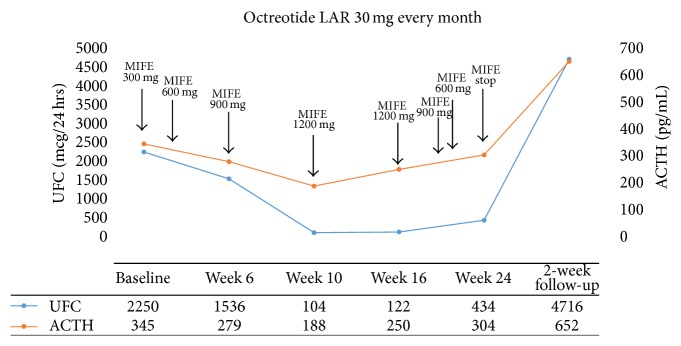
UFC and ACTH concentrations during treatment with mifepristone and octreotide LAR. ACTH denotes adrenocorticotropin hormone, LAR long-acting release, MIFE mifepristone, and UFC urinary-free cortisol. To convert the values for UFC to nanomoles per 24 h, multiply by 2.76. To convert the values for ACTH to picomoles per liter, multiply by 0.22.

A 30-year-old man presented with weight gain, hypertension, diabetes, proximal muscle weakness, and nephrolithiasis. He developed moon facies, abdominal striae, and disproportionate supraclavicular and dorsocervical fat pads. Endocrine testing and imaging studies revealed a pancreatic NET with involvement of the inferior vena cava and other local structures. After resection of the tumor, the postoperative ACTH was not suppressed; however, cushingoid features improved significantly. Approximately 2 years later, symptoms of EAS recurred. Ketoconazole and chemotherapy were started but were not successful in resolving hypercortisolemia. Three months later, octreotide LAR was initiated, and the dose was gradually increased to 30 mg every month. A partial biochemical response was noted (ACTH decreased from 517 pg/mL (113.7 pmol/L) to 345 pg/mL (75.9 pmol/L)), but the patient's symptoms of EAS were not controlled. After 3 months of therapy with octreotide LAR, the patient was enrolled in a 24-week, phase 3 clinical trial of mifepristone for inoperable hypercortisolemia (clinicaltrials.gov identifier: NCT00569582 [17]). Prior to the start of mifepristone, baseline urinary-free cortisol (UFC) was 2250 mcg/24 hours (6207 nmol/24 hours) and ACTH was 345 pg/mL (75.9 pmol/L). Late-night salivary cortisol (1.71 mcg/dL (47.2 nmol/L)) and serum cortisol (46 mcg/dL (1256 nmol/L)) were also elevated (Table 1). At the time of enrollment, the patient had overtly cushingoid features, including moon facies, plethora, and enlarged dorsocervical and supraclavicular fat pads; purple striae; bruising; edema; and proximal muscle weakness that was so severe that he was unable to rise from a chair without use of his hands. He also had ongoing diabetes, depression, and hypertension associated with hypokalemia. Mifepristone was initiated at a daily dose of 300 mg and gradually increased to 1200 mg per protocol. The patient continued to receive octreotide LAR throughout the duration of the trial. By week 4, insulin therapy was discontinued and by week 12, his cushingoid features essentially resolved. In addition to clinical improvement, a dramatic decrease in cortisol and ACTH was noted during therapy with mifepristone and octreotide LAR (Figure 1, Table 1). At week 20, mifepristone was briefly stopped for significant fatigue, low appetite, and nausea. Mifepristone was then resumed at a daily dose of 900 mg and 1 week later reduced to 600 mg; no changes were made to octreotide LAR dose. At week 24, his UFC and ACTH levels were 434 mcg/24 hours (1198.7 nmol/24 hours) and 304 pg/mL (66.9 pmol/L), respectively, and mifepristone was stopped per study protocol. During withdrawal of mifepristone, the cortisol and ACTH rose, and 12 days after mifepristone was stopped, clinical signs and symptoms of EAS returned. After 2 weeks, his UFC and ACTH increased to 4716 mcg/24 hours (13016 nmol/24 hours) and 652 pg/mL (143.4 pmol/L), respectively (Figure 1, Table 1). Mifepristone was resumed for an additional 12-month extension period. Octreotide LAR was discontinued after 2 months and the patient continued with mifepristone for control of his CS-related symptoms. The collection of cortisol and ACTH data was less frequent during the extension study. At the time octreotide was discontinued, the patient's ACTH and serum cortisol were 652 pg/mL (143.4 pmol/L) and 67.8 mcg/dL (1871 nmol/L), respectively. After 12 months in the extension phase, substantial increases in ACTH (3738 pg/mL (822.4 pmol/L)), serum cortisol (135.2 mcg/dL (3732 nmol/L)), and UFC (10716.5 mcg/24 hours (29577.5 nmol/24 hours)) were observed.

## 3. Discussion

This case describes a patient with pancreatic NET associated with EAS, in whom treatment with the somatostatin analog octreotide became much more effective in controlling cortisol and ACTH after the addition of GR antagonist therapy with mifepristone. Several studies have demonstrated a relationship between GR sensitivity and response to somatostatin analogs [13, 15, 18]. Corticotrope adenomas contain multiple somatostatin receptors, primarily SST5 and lower levels of SST2 [19]. Using murine corticotrope tumor cells, van der Hoek et al. demonstrated a dexamethasone-dependent inhibitory effect of octreotide treatment targeting SST2 that was not found with analogs that targeted primarily SST5 [15]. This result suggests that analogs targeting SST2 are particularly susceptible to glucocorticoid-mediated downregulation, which also might explain the lower SST2 expression found in human corticotrope adenomas and the frequent lack of response to octreotide in patients with Cushing's disease (CD) [14].

Ferrau et al. studied whether a brief treatment with mifepristone modulates the response to acute octreotide administration in 5 patients with CD [18]. This study showed that brief mifepristone pretreatment does not modify ACTH and cortisol response to acute octreotide administration in patients with CD. However, the authors noted that, regardless of mifepristone treatment, decreases in ACTH and cortisol after acute injection of octreotide were observed in patients with lower cortisol levels following dexamethasone suppression testing. Together these results suggest that an intact glucocorticoid signaling pathway within these cells is required for downregulation of SST2.

This model could explain some of the variability in response to SST2 analogs among the various tumor types associated with EAS. In vitro studies in small-cell lung cancer cell lines demonstrated defects in GR function that could lead to glucocorticoid resistance [20, 21]. de Bruin et al. have shown a dexamethasone dose-dependent downregulation of SST2 expression in the human pancreatic NET cell line BON and the medullary thyroid carcinoma cell line TT, which disappeared after treating these cells with mifepristone [13]. However, the authors reported no glucocorticoid-mediated effects in DMS cells from a small-cell lung cancer line with severe glucocorticoid resistance.

At least 20% to 30% of patients with EAS will suppress plasma and urinary steroids to less than 50% of baseline values during high-dose dexamethasone suppression testing [22]. These patients are clinically and hormonally difficult to differentiate from patients with CD, particularly if the tumor is not localized. With the development of targeting radionuclide diagnostics and therapeutics using somatostatin analogs (theranostics), an additional role of mifepristone might be as a “radiosensitizing” agent. Ejaz et al. reported 2 patients with EAS and occult tumor sources despite multiple imaging attempts whose bronchial NETs were localized via octreotide scintigraphy only after receiving treatment with mifepristone for several weeks [2]. Of note, only 1 of the 2 patients was found to be responsive to high-dose dexamethasone testing. Therefore, additional data are required to determine whether a positive response to 1 mg of dexamethasone in patients with CD or 8 mg in patients with EAS can predict the response to somatostatin analogs targeting the SST2.

In this case, the addition of mifepristone to ongoing octreotide LAR led to a substantial reduction in ACTH and cortisol in our patient with previously resistant EAS associated with severe hypercortisolemia. This effect was lost upon discontinuation of mifepristone after 24 weeks of treatment. Of note, the reduction in ACTH during mifepristone cotreatment was not as pronounced as the observed reduction in cortisol. An assessment of ACTH precursors (proopiomelanocortin and pro-ACTH), which can be markedly elevated in patients with EAS [23, 24], would have provided additional insight. However, measuring precursors and biologically active ACTH would have required separation via chromatography, which was not performed, nor did we have access to patient tumor tissue to assess SST receptor expression. Nonetheless, to our knowledge, this is the first clinical case report that demonstrates a relationship between GR antagonism with mifepristone and increased therapeutic efficacy of the somatostatin analog octreotide, consistent with upregulation of somatostatin receptors previously downregulated by hypercortisolemia.

## Figures and Tables

**Table 1:** Biochemistry evaluations during treatment with mifepristone^*∗*^.

Test (normal range)	Baseline (before MIFE)	Week 6	Week 10	Week 16	Week 24	2-week follow-up (off MIFE)
ACTH, pg/mL (7–50 pg/mL)	345	279	188	250	304	652

UFC, mcg/24 h (2.0–42.4 mcg/24 h)	2250	1536	104	122	434	4716

Serum cortisol, mcg/dL (8 AM, 4.0–22.0 mcg/dL)	46	41	31	31	37	68

Late-night salivary cortisol, mcg/dL (10 PM-11 PM, ≤0.09 mcg/dL)	1.71	2.18	0.56	0.73	1.49	4.91

^*∗*^Patient continued to receive octreotide LAR 30 mg every month throughout the study. ACTH denotes adrenocorticotropin hormone, LAR long-acting release, MIFE mifepristone, and UFC urinary-free cortisol. To convert the values for UFC to nanomoles per 24 h, multiply by 2.76. To convert the values for ACTH to picomoles per liter, multiply by 0.22. To convert the values for serum cortisol and late-night salivary cortisol to nanomoles per liter, multiply by 27.6.
